# Scapulothoracic Disarticulation of the Right Upper Limb After a Car-to-Obstacle Collision: A Case Report

**DOI:** 10.7759/cureus.116815

**Published:** 2026-09-24

**Authors:** Ana Carolina Augustini Farinazzo, Vinicius Rodrigues Pasetto, Bernardo Cassilha, Alice Rocha, Julya Felber, Diego Akyo dos Santos, Mariana Muraro, Fernando Ferraz Faria

**Affiliations:** 1 Orthopedics and Traumatology, Hospital Universitário Cajuru, Curitiba, BRA

**Keywords:** forequarter amputation, high-energy trauma, polytrauma, traumatic amputation, traumatic disarticulation, upper limb

## Abstract

This report details the case of a 26-year-old man, victim of a car-to-barrier collision followed by a rollover. The patient, found conscious, was intubated by the pre-hospital team and admitted in severe hypovolemic shock, with extensive injury to the shoulder and right upper limb. After hemodynamic stabilization, the team performed bilateral chest drainage, a massive transfusion protocol, and a forequarter amputation of the right upper limb with ligation of the subclavian vessels, in a joint procedure by the orthopedic and vascular surgery teams. Postoperatively, he remained in the intensive care unit (ICU), developing ventilator-associated pneumonia, treated with meropenem and vancomycin. After 10 days, the patient was transferred to a ward. He was discharged from the hospital after 15 days of hospitalization and continued with outpatient follow-up, where he presented with phantom limb pain initially treated with gabapentin and controlled with physiotherapy. In the end, he was definitively discharged with good healing, pain improvement, and favorable clinical evolution.

## Introduction

Traffic accidents constitute a significant public health problem, with substantial clinical, social, and economic repercussions, especially among young men. In Paraná, a state in Brazil, between 2022 and 2023, 6060 young people aged 20 to 29 were hospitalized for traffic injuries in public hospitals or those affiliated with the Unified Health System (SUS), and 1119 died as a result of these accidents, according to the State Health Department. Hospitalizations in this age group represented an approximate cost of R$ 10.2 million for the system in that period alone [1].

Traumatic upper limb amputations constitute serious and potentially disabling injuries, but their occurrence is relatively uncommon in the trauma victim population. A study conducted using the United States trauma database showed that between 2009 and 2012, 1386 patients underwent major upper limb amputation due to trauma, corresponding to an incidence of 46 cases per 100,000 hospital admissions for trauma. The study also defined the most frequent amputation levels, with transhumeral amputation occurring in 35% of cases, followed by forearm amputation (30%) and hand amputation (14%), with 33.1% of cases resulting from accidents where the patient was a passenger in a motor vehicle [2].

Although traumatic disarticulations, which is the separation of two bones at their connecting joint, are more frequent in lower limbs, as seen previously, their occurrence in upper limbs represents a specific challenge, especially in the context of high-energy trauma. The decision between preserving or disarticulating a limb depends on multiple factors, including the patient's hemodynamic stability, the extent of vascular injury, soft tissue involvement, and the functional viability of the limb. Tools such as the Mangled Extremity Severity Score (MESS) assist in decision-making, although clinical judgment remains fundamental. In polytrauma patients with a critical physiological state, limb preservation may be contraindicated [3].

Another possibility would be limb replantation, however, despite advances in microsurgical and reconstructive techniques, this procedure after traumatic upper limb amputation remains indicated only in selected cases. A North American analysis conducted between 2008 and 2017 identified 2305 cases of traumatic upper limb amputations, of which only 22.3% underwent attempted replantation, with the vast majority (87.4%) at the forearm level [4]. These data demonstrate that reimplantation is performed in a small portion of traumatic amputations, being more commonly performed in lesions at the forearm level, while more proximal amputations present greater complexity for replantation, thus making the decision for definitive amputation more common.

This report describes a case of traumatic disarticulation of the right upper limb resulting from a high-energy automobile accident, emphasizing the initial care, surgical strategy, hospital complications, and outpatient follow-up, contributing to the discussion on decision-making criteria, surgical approach, and interdisciplinary management in situations of severe trauma and in the management of these rare injuries.

## Case presentation

A 26-year-old male patient was admitted to the emergency room of a public trauma referral hospital in Curitiba-Paraná, Brazil, after being involved in a car collision with a barrier followed by a rollover. He was found conscious at the scene, but underwent orotracheal intubation (OTI) by the pre-hospital care team due to a decreased level of consciousness.

Upon admission, he presented with unmeasurable blood pressure, tachycardia, and hemodynamic instability, requiring mechanical ventilation, without initial need for vasoactive drugs. Physical examination revealed extensive injury to the shoulder and right upper limb, with bone exposure (scapula) and extensive soft tissue injury (Figure 1).

**Figure 1:**
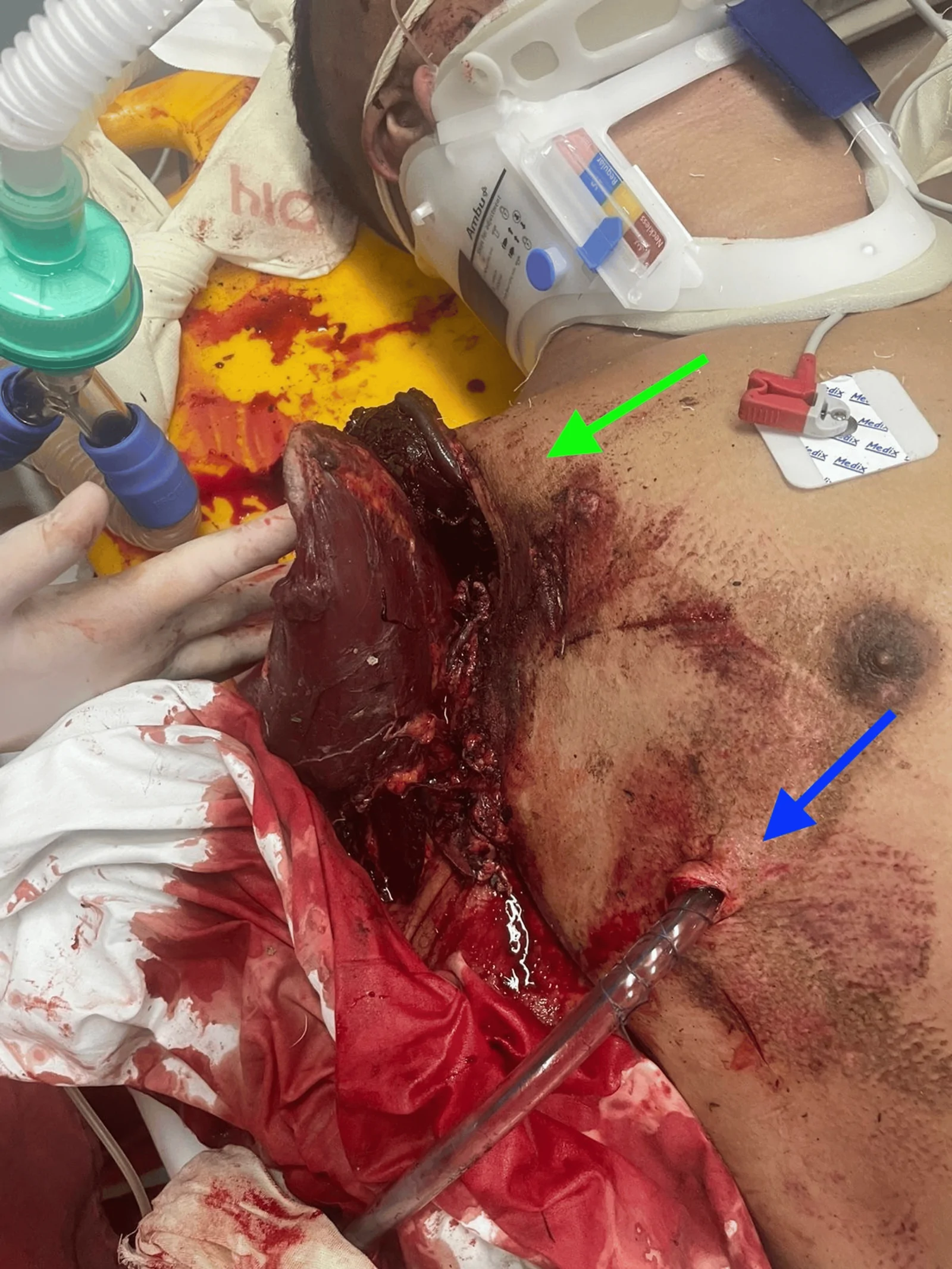
Image of the patient upon admission to the emergency room (personal collection). The green arrow indicates the injury at the time of admission, and the blue arrow indicates the right chest drain.

In the emergency room, bilateral closed chest drainage was performed due to the absence of bilateral pleural sliding during the Focused Assessment with Sonography in Trauma (FAST) exam in the emergency room, and a massive transfusion protocol was initiated by general surgery, with the administration of 3 units of packed red blood cells, 3 units of platelets, 3 units of plasma, and 1 gram of tranexamic acid. Following stabilization, the patient underwent additional scans, revealing an acute subdural hematoma, and spinal injuries were ruled out. The patient was subsequently taken to the operating room (Figure 2).

**Figure 2:**
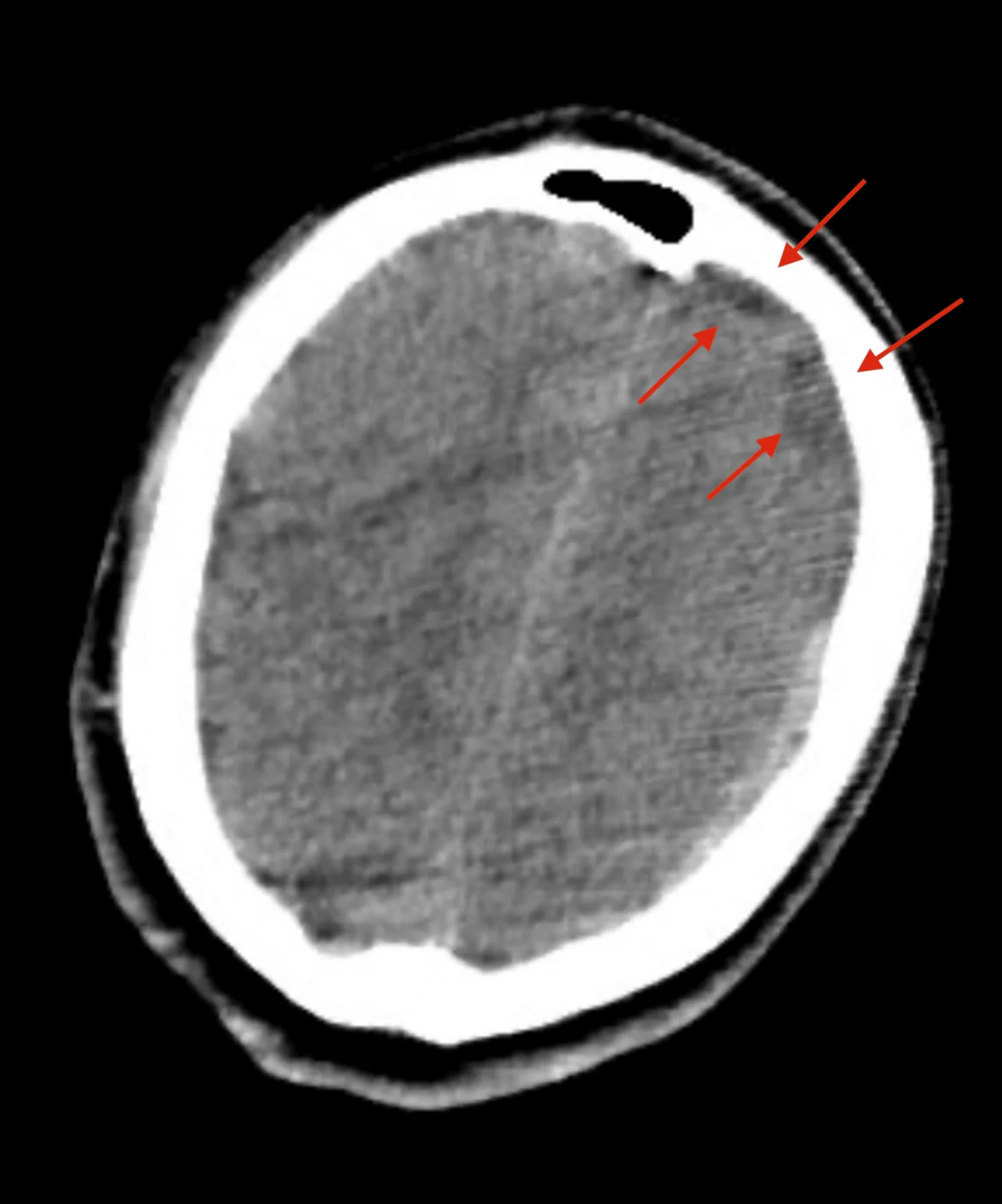
Computed tomography of the skull showing a left frontoparietal subdural hematoma. The red arrows indicate the left frontoparietal subdural hematoma.

In the operating room, the patient underwent drainage of a large subdural hematoma and placement of an intracranial pressure monitoring catheter by the neurosurgery team. An exploratory laparotomy was also performed by the general surgery team, revealing a moderate amount of blood in the abdominal cavity, a hepatic laceration without active bleeding, and a non-expanding right-sided retroperitoneal hematoma. With no other injuries observed, hemostasis was reviewed, the cavity was lavaged, and the incision was closed in layers.

During the intraoperative period, after positioning in the operating room, it was observed that the right upper limb was connected to the thorax only by the posterosuperior portion of the skin, the teres minor muscle, and part of the brachial plexus. The vascular surgery team ligated the subclavian artery and vein. The orthopedic team then proceeded with the disarticulation of the limb (Figure 3), associated with resection of the deltoid muscle, osteotomy of the distal half of the clavicle, and tenodesis of the pectoralis major muscle to the clavicle; these procedures were performed to enable skin closure and to maintain a soft-tissue cushion for the amputation stump. Replantation was contraindicated due to massive destruction of soft tissues, vascular and neurological structures. At the end, a control chest X-ray was performed (Figure 4).

**Figure 3:**
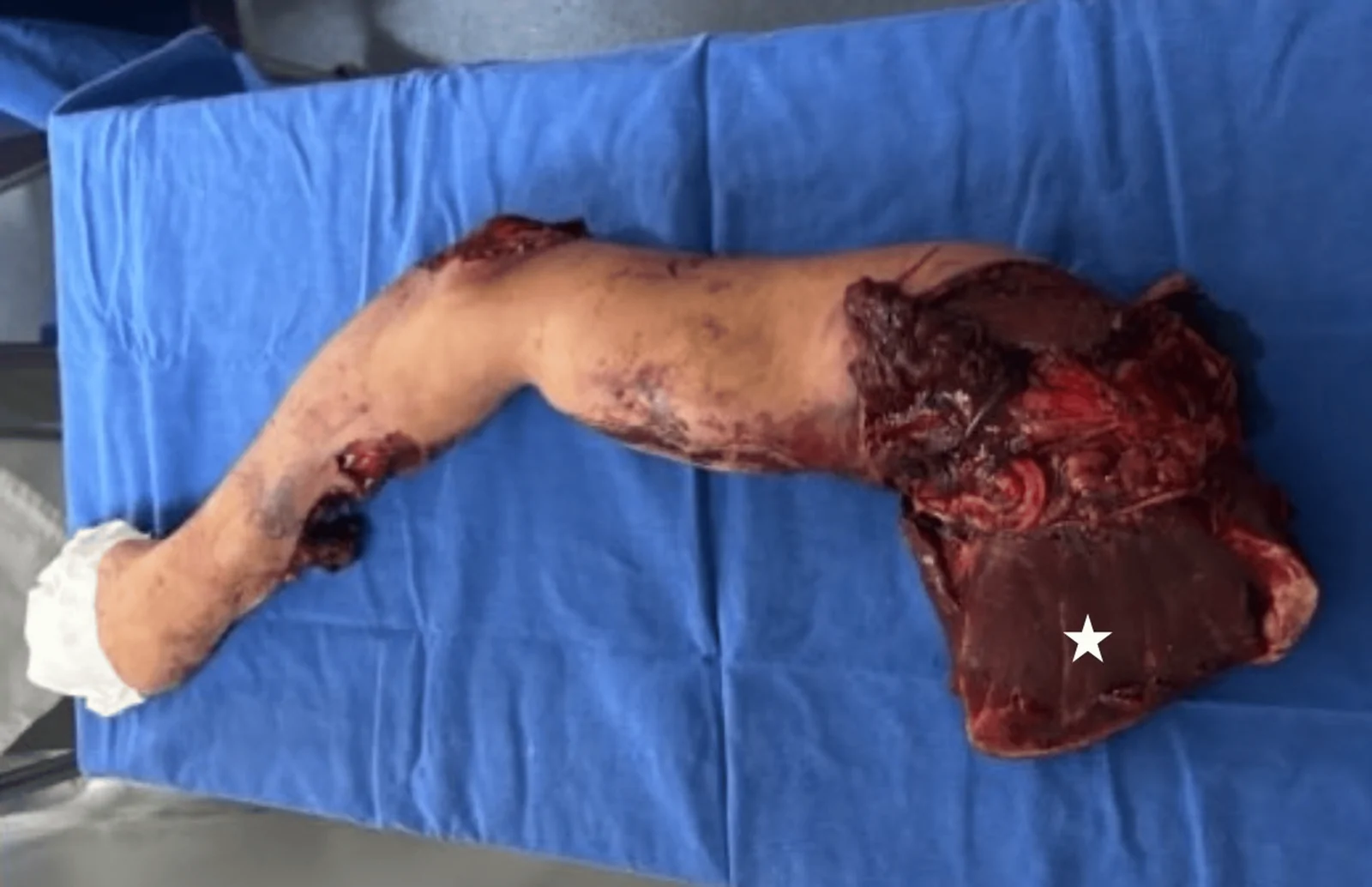
Image of the disarticulated right limb in the operating room (personal collection). The star indicates the scapula.

**Figure 4:**
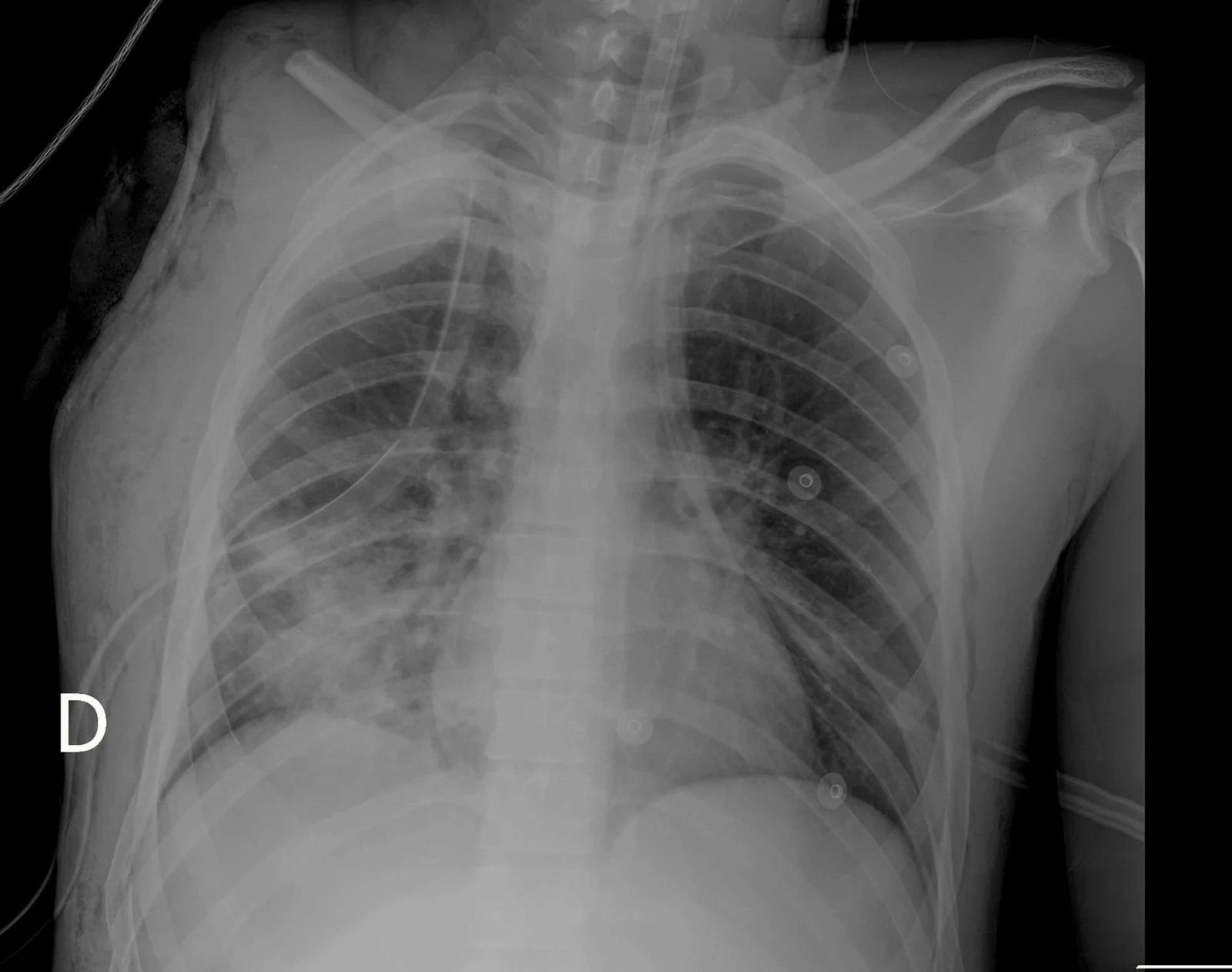
Chest X-ray in AP view after disarticulation of the right upper limb.

The patient was admitted to the intensive care unit (ICU) in the immediate postoperative period, where he developed ventilator-associated pneumonia (VAP) and was treated with meropenem 1g every 8 hours and vancomycin 1g every 12 hours, initiated and maintained for six days. After 10 days, he showed clinical and laboratory improvement and was transferred to a ward, where he remained under the care of specialists. He was discharged from the hospital after 15 days of hospitalization and referred to specialist outpatient clinics and a rehabilitation hospital.

At discharge, the patient was prescribed amitriptyline 75mg nightly and gabapentin 600mg every 8 hours to control phantom limb pain, in addition to diazepam 5mg for three weeks with gradual tapering. The patient was referred for outpatient consultations with the orthopedics, vascular surgery, neurosurgery, and general surgery teams. Other specialties monitored the patient for potential procedure-related complications; however, none occurred, and the patient was discharged following full recovery.

During the initial outpatient consultations with the orthopedics team, the patient still reported phantom limb pain; gabapentin and amitriptyline were continued, and the patient was referred for physiotherapy. During follow-up, the patient reported progressive improvement in phantom limb pain and had no complaints of dizziness or neurological changes. After seven months, the patient presented with good wound healing, without significant pain complaints, and was discharged from follow-up. The patient has not yet been fitted with a prosthesis for the limb; however, he is able to perform activities of daily living and has adapted to the amputation.

## Discussion

Traumatic disarticulations are injuries that predominantly affect men of working age, contributing to significant functional loss, impaired quality of life, and work disability, with a high economic impact on the country [5]. Unlike lower limb disarticulations, where the most common etiology is peripheral vascular disease, trauma is the main cause of upper limb disarticulation [6].

In general, traumatic disarticulations result from high-energy mechanisms, such as car accidents, explosions, and occupational accidents involving machinery [5,6]. This poses additional challenges due to the anatomical complexity of the shoulder girdle, proximity to large vessels, and high risk of hemorrhagic shock, as in the present case where the decision for immediate disarticulation was motivated by the patient's critical physiological condition and the impossibility of preserving the limb, given the massive destruction of soft tissues and proximal vascular injury.

According to the Advanced Trauma Life Support (ATLS) protocol of the American College of Surgeons' trauma committee, hemorrhage is the most preventable cause of mortality in trauma. External bleeding, especially from the proximal segment of the limbs, is life-threatening and must be controlled immediately. Initial care for the polytrauma patient should follow a systematic approach, starting with the airway (A), followed by respiratory pattern (B), circulatory status (C), neurological assessment (D), and finally exposure with hypothermia control (E), identifying and treating potentially fatal conditions in order of priority, allowing for rapid and targeted interventions [6].

In this context, due to extensive soft tissue injury and high-energy trauma, the patient was referred in a state of hemorrhagic shock, and measures were taken to control massive bleeding, perform fluid resuscitation, and preserve life. Upper limb trauma can be classified according to the Mangled Extremity Severity Score (MESS). Although primarily designed for lower-limb amputations, it can be adapted for use on upper limbs. This tool can assist in assessing the severity of extremity injuries, although its ability to predict the need for amputation is limited and should not replace clinical judgment; high scores may indicate a higher probability of amputation. In this case, the patient presented a MESS score of 9 points. The patient scores 4 points due to the high-energy trauma, 3 points for limb ischemia - presenting with a cold, paralyzed, and insensate limb - and 2 points for persistent hypotension. The patient receives no points for age, and the ischemia time was less than 6 hours, resulting in a total score of 9 points. As previously stated, the MESS should not be used as an isolated instrument for indicating amputation; however, the indication for disarticulation was also based on hemorrhagic shock, massive soft tissue destruction, proximal vascular injury, neurological injury, anatomical inviability of the limb, and impossibility of replantation. Being objective and easy to apply, this scale also plays a supporting role in decision-making for cases of traumatic amputation - always in conjunction with clinical judgment and tailored to each patient and each trauma [7].

The possibility of replantation should be considered in traumatic upper limb amputations; however, its indication depends on the amputation level, injury mechanism, ischemia time, clinical conditions and vascular and nerve viability [4]. In the reported case, extensive soft tissue injury, neurovascular lesions and patient instability made reimplantation unfeasible.

Traumatic upper limb disarticulation is associated with high physical and psychosocial morbidity, significantly impacting quality of life, functionality, and return to work. In addition to the risks inherent in the surgical procedure - such as wound infection, necrosis or dehiscence of the stump, hematoma and seroma formation, intense pain, deep vein thrombosis, and respiratory complications - traumatic amputation poses complex challenges in the psychosocial sphere and in rehabilitation. Conditions such as depression and post-traumatic stress disorder (PTSD) are frequently observed among these victims. The United States military population has been extensively studied in this context: an American meta-analysis revealed that at least one in three amputees presented with depression, while one in four was diagnosed with PTSD [8]. Studies focused exclusively on the civilian population demonstrate even higher rates of these conditions [9]. It is therefore essential that the surgical team share the care of this type of patient with professionals in psychology, psychiatry, occupational therapy and physiotherapy, promoting better rehabilitation and return to work activities [5,10].

This case is relevant because it demonstrates a favorable clinical evolution in a high-energy trauma with the implementation of protocols for rapid and coordinated care of traumatic amputations in referral centers, as well as the importance of integration between specialties to optimize functional outcomes and long-term quality of life.

The patient in this case, despite experiencing phantom pain post-operatively, responded well to treatment, which included medication and physical therapy, showing improvement in phantom pain, good healing, and discharge after seven months.

The notable feature of the reported case lies in the extremely proximal nature of the lesion, associated with extensive damage to soft tissues and neurovascular structures. These conditions limited the possibility of limb reconstruction and necessitated disarticulation as a measure to control the injury and preserve life.

## Conclusions

This case highlights the challenges of managing traumatic disarticulation of the right upper limb in high-energy trauma, emphasizing the need for a rapid and coordinated approach, according to the Advanced Trauma Life Support (ATLS), to preserve life and demonstrating how traffic accidents represent a major public health challenge. The decision to disarticulate the upper limb was necessary to control the injury and clinical instability of the patient, contributing to a favorable outcome, considering the high-energy trauma with the patient's unfavorable clinical conditions, the extensive destruction of the limb, the infeasibility of reimplantation, and prioritizing hemorrhage control and preservation of life.

This case reinforces the importance of multidisciplinary and longitudinal follow-up, involving not only orthopedic and vascular surgeons, but also physiotherapy, psychotherapy, and occupational therapy, in order to promote an adequate rehabilitation process and, eventually, quality of life, optimizing outcomes in situations of extreme severity. Although this involves a single case in which management and clinical approach were evaluated and demonstrated, caution should be exercised regarding generalizations; each patient should be evaluated individually to ensure the best decision-making.
